# Supplementary material for: Rapid ecosystem-scale consequences of acute deoxygenation on a Caribbean coral reef
Source: Nat Commun. 2021 Jul 26;12:4522. doi: 10.1038/s41467-021-24777-3 (PMC8313580; doi:10.1038/s41467-021-24777-3)
Supplement: Supplementary file 1 — Supplementary Information [file 41467_2021_24777_MOESM1_ESM.pdf]

Rapid ecosystem-scale consequences of acute deoxygenation on a Caribbean coral reef

Electronic Supplementary Material

Maggie D. Johnson[1,2,3,7*], Jarrod J. Scott[1,7], Matthieu Leray[1,7], Noelle Lucey[1,7], Lucia M. Rodriguez Bravo[1,4], William L. Wied[1,5], Andrew H. Altieri[1,6]

[1]Smithsonian Tropical Research Institute, Balboa, Ancon, República de Panamá
[2]Tenenbaum Marine Observatories Network, Smithsonian Institution, Edgewater, MD, USA

Present Address

[3]Biology Department, Woods Hole Oceanographic Institution, Woods Hole, MA, USA
[4]Facultad de Ciencias Marinas, Universidad Autónoma de Baja California, Ensenada, Mexico
[5]Department of Biological Sciences, Center for Coastal Oceans Research, Florida International University, FL, USA
[6]Department of Environmental Engineering Sciences, University of Florida, Gainesville, FL, USA

[7]These authors contributed equally.

[*]Corresponding author: magjohnson@gmail.com

Contents

Overview 2
Content Description 2
Data & Code Availability 2
Supplementary Tables 3
Supplementary Figures 3
Supplementary Figure 1 3
Supplementary Figure 2 4
Supplementary Figure 3 5
Supplementary Figure 4 6
Supplementary Figure 5 7
Supplementary Data 8
Supplementary Data 1 8
Supplementary Data 2 8

Supplementary Data 3 9
Supplementary Data 4 9

Overview

This document contains Supplementary tables and figures for the manuscript. In several cases, tables were too large to fit in this document and are instead provided as additional **Supplementary Data** files (see below for further details).

An HTML version of this file, including all Supplementary Data, can be found on the project website at https://hypocolypse.github.io/som.html.

Content Description

Data & Code Availability: DOI and accession numbers for study related data.
Supplementary Table 1: Primer sequences for 16S rRNA gene amplification.
Supplementary Figure 1: Environmental conditions during the acute deoxygenation event.
Supplementary Figure 2: Photophysiology of *Agaricia* during the acute deoxygenation event.
Supplementary Figure 3: Percent of total available coral substrate by genus and health condition.
Supplementary Figure 4: Phylogenomic analysis of MAG02.
Supplementary Figure 5: Phylogenomic analysis of MAG04.
Supplementary Data 1: Differentially abundant ASVs.
Supplementary Data 2: KEGG module estimations for MAGs.
Supplementary Data 3: Metadata of genomes in MAG02 phylogenomic analysis.
Supplementary Data 4: Metadata of genomes in MAG04 phylogenomic analysis.

Data & Code Availability

We provide additional data products and code through online repositories. For further details, please see the Data Availability page on the project website at https://hypocolypse.github.io/data-availability.html.

- https://doi.org/10.25573/data.c.5025362.v1: Figshare collection containing all data and data products.
- https://zenodo.org/badge/latestdoi/268354818: Zenodo archive containing reproducible workflows in RMarkdown format.
- https://doi.org/10.25573/data.11819745.v4: Raw 16S rRNA data for each sample (before removing primers).
- https://www.ebi.ac.uk/ena/browser/view/PRJEB36632: Study accession number PRJEB36632 (ERP119845) sequencing data deposited at the European Nucleotide Archive.
- https://www.ebi.ac.uk/ena/browser/view/ERS4291994-ERS4292031: Sample accession numbers (ERS4291994-ERS4292031) for trimmed 16S rRNA data (primers removed) deposited at the European Nucleotide Archive.
- https://www.ebi.ac.uk/ena/browser/view/ERS4578390-ERS4578393: Sample accession numbers (ERS4578390-ERS4578393) for the metagenomic data from the four water column samples deposited at the European Nucleotide Archive.

Supplementary Tables

Supplementary Table 1. Primer sequences for 16S rRNA gene amplification.

Primer name	Primer sequence
515F	GTGYCAGCMGCCGCGGTAA
926R	CCGYCAATTYMTTTRAGTTT

Supplementary Figures

Supplementary Figure 1

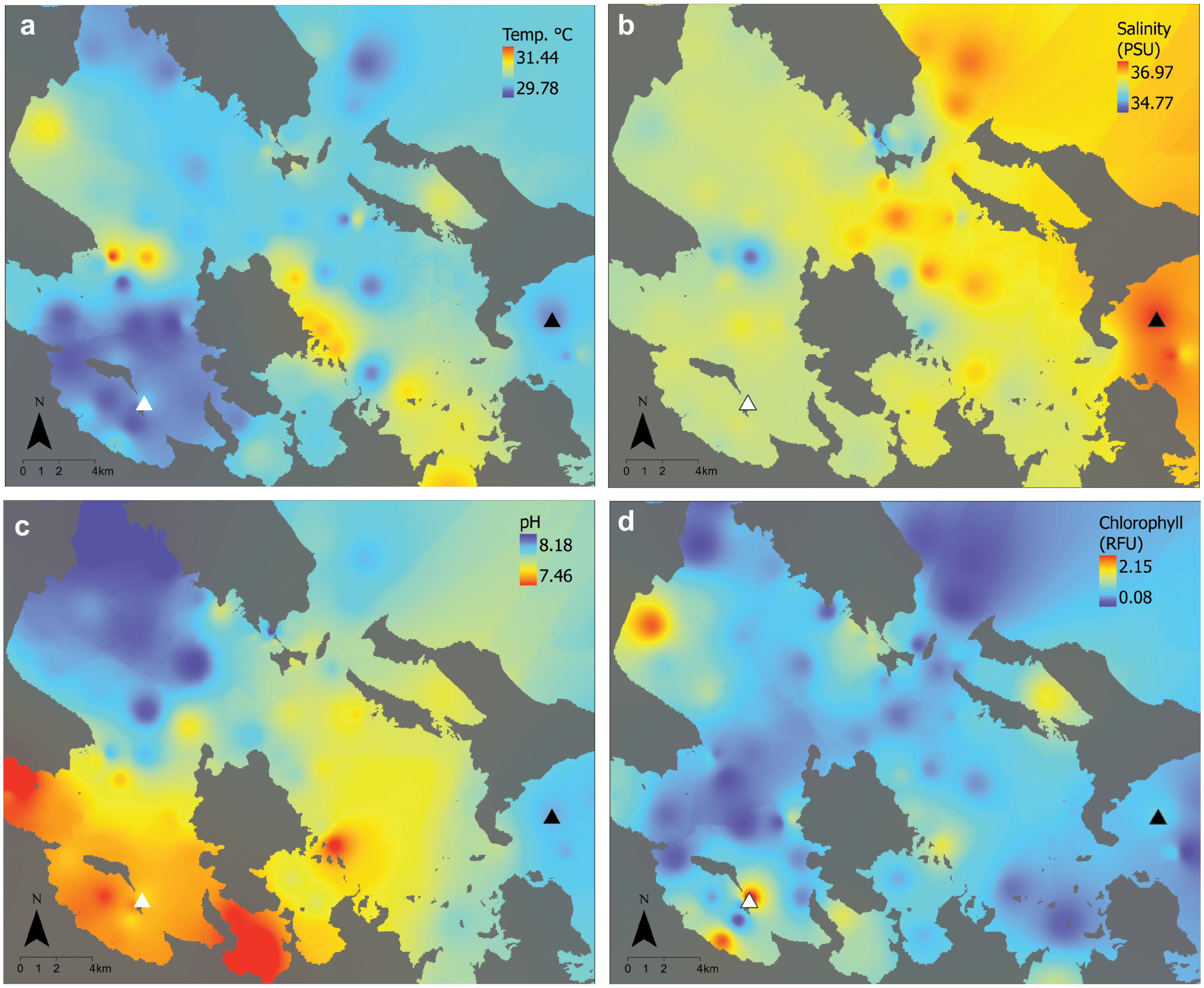

Supplementary Fig. 1. Environmental conditions during the 2017 acute deoxygenation event in Bocas del Toro, Panamá. Maps of environmental conditions across Bahía Almirante six days after initial detection of the hypoxic event, with locations of the impacted site (white triangle) and the non-impacted reference site (black triangle). **a**) Temperature is in ºC, **b**) salinity is in practical salinity units (PSU), **c**) pH is presented on the NBS scale, and **d**) chlorophyll concentrations are presented as relative fluorescence units (RFU).

Supplementary Figure 2

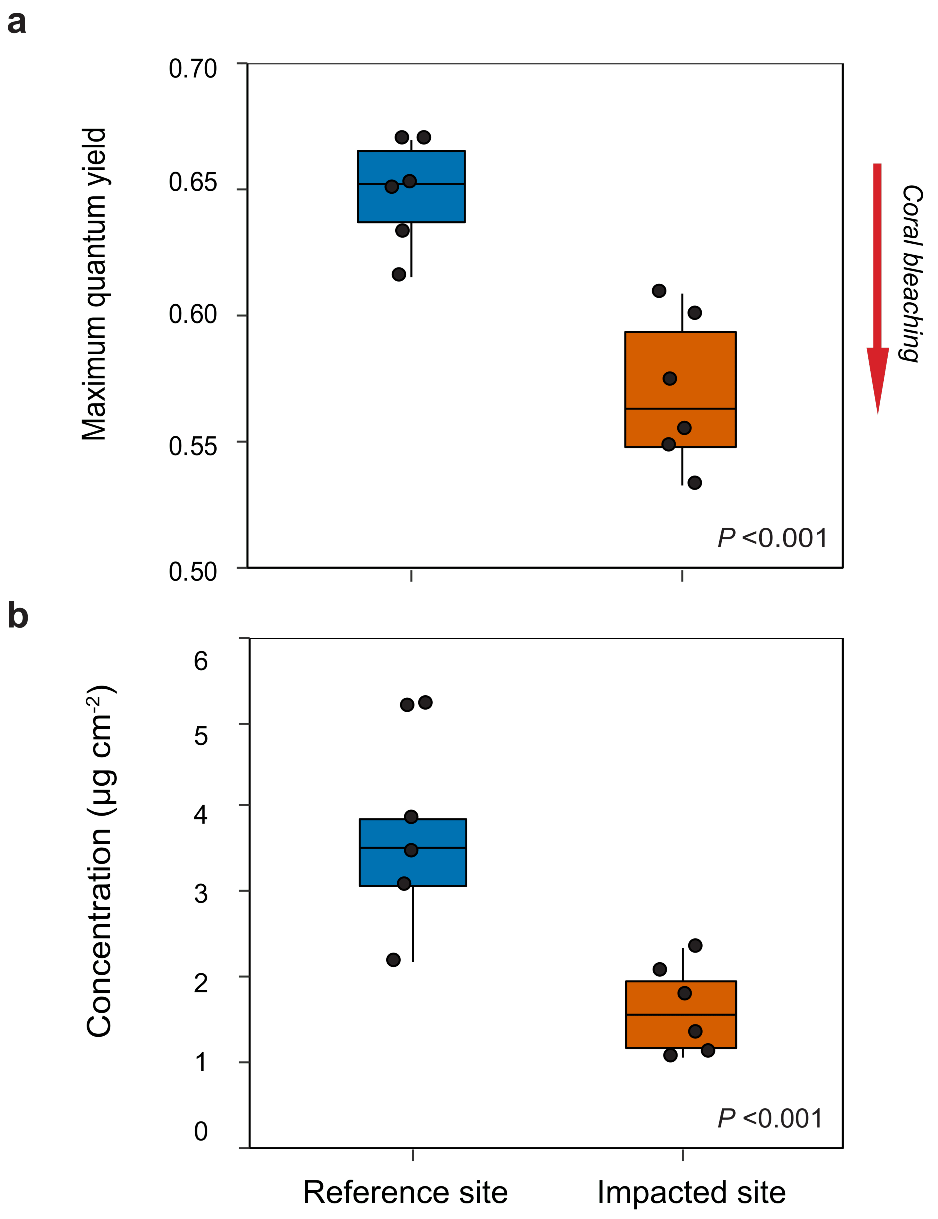

Supplementary Fig. 2. Photophysiology of *Agaricia* during the acute deoxygenation event. Box plots for *Agaricia tenuifolia* photophysiology from corals collected at the impacted (vermillion) and reference (blue) sites during the deoxygenation event. The center line indicates the median, the box limits the interquartile range, and the whiskers the minimum and maximum for the data. In each plot the colony means are overlaid as black dots (N = 6 biologically independent colony replicates). Differences between sites were evaluated with linear mixed effects models with site as a fixed effect and colony as a random effect. **a**) Maximum quantum yield ($P = 1.156e^{-6}$) and **b**) chlorophyll *a* concentrations were significantly lower at the impacted site ($P = 6.851e^{-07}$). Lower quantum yield and chlorophyll concentrations are indicative of coral bleaching (red arrow).

Supplementary Figure 3

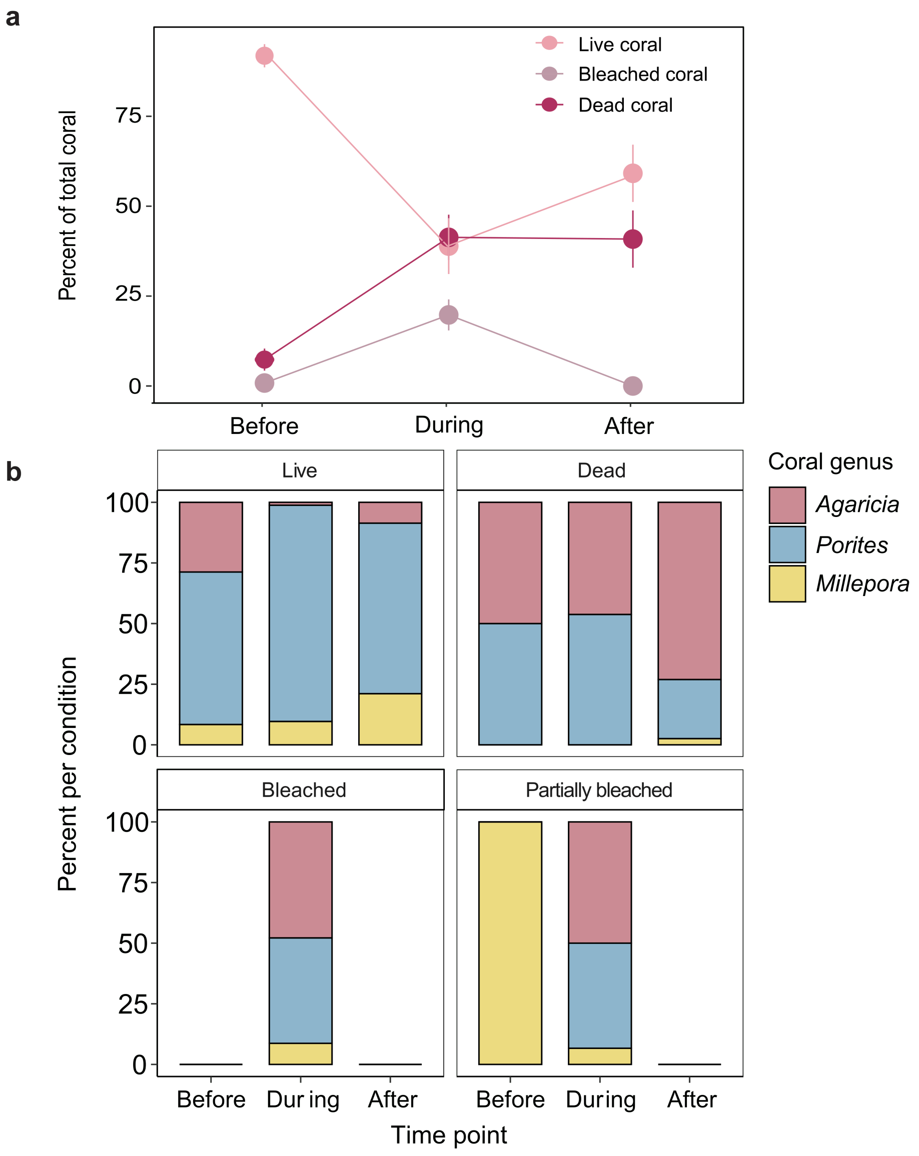

Supplementary Fig. 3. Percent of total available coral substrate by genus and health condition. **a**) Mean percent (± SE) of live, bleached (including partially bleached), and dead corals at each time point relative to the total amount of coral substrate available at the impacted site ($N = 11$ independent plot replicates per time point). There were no significant changes in the benthic community at the reference site over the duration of the study. **b**) Stacked bar charts of the percent of the dominant coral genera per health condition before, during, and after the hypoxic event at the impacted site.

Supplementary Figure 4

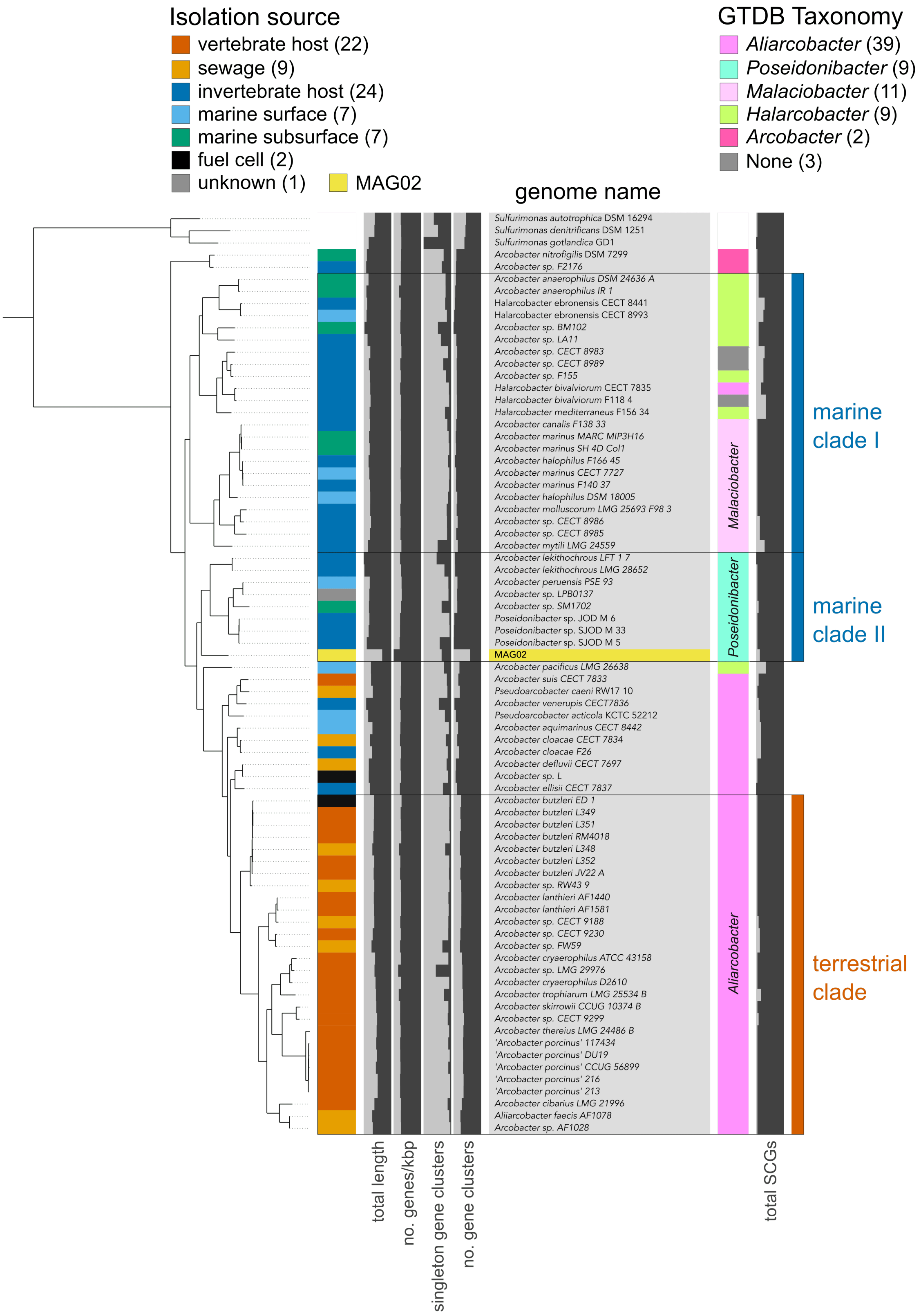

Supplementary Fig. 4. Phylogenomic analysis of MAG02. Phylogenomic placement of MAG02 against a manually curated collection of 72 *Arcobacter* group genomes. The tree was rooted with three *Sulfurimonas* genomes. The colored bar on the left indicates the broad habitat category, assigned based on the host and isolation source retrieved from NCBI's BioSample database for each genome. Values in parentheses indicate the number of genomes per category. Results of taxonomy estimates for each genome are shown on the right, based on a collection of 23 ribosomal genes from the Genome Taxonomy Database (GTDB). See Supplementary Data 3 for information on source data.

Supplementary Figure 5

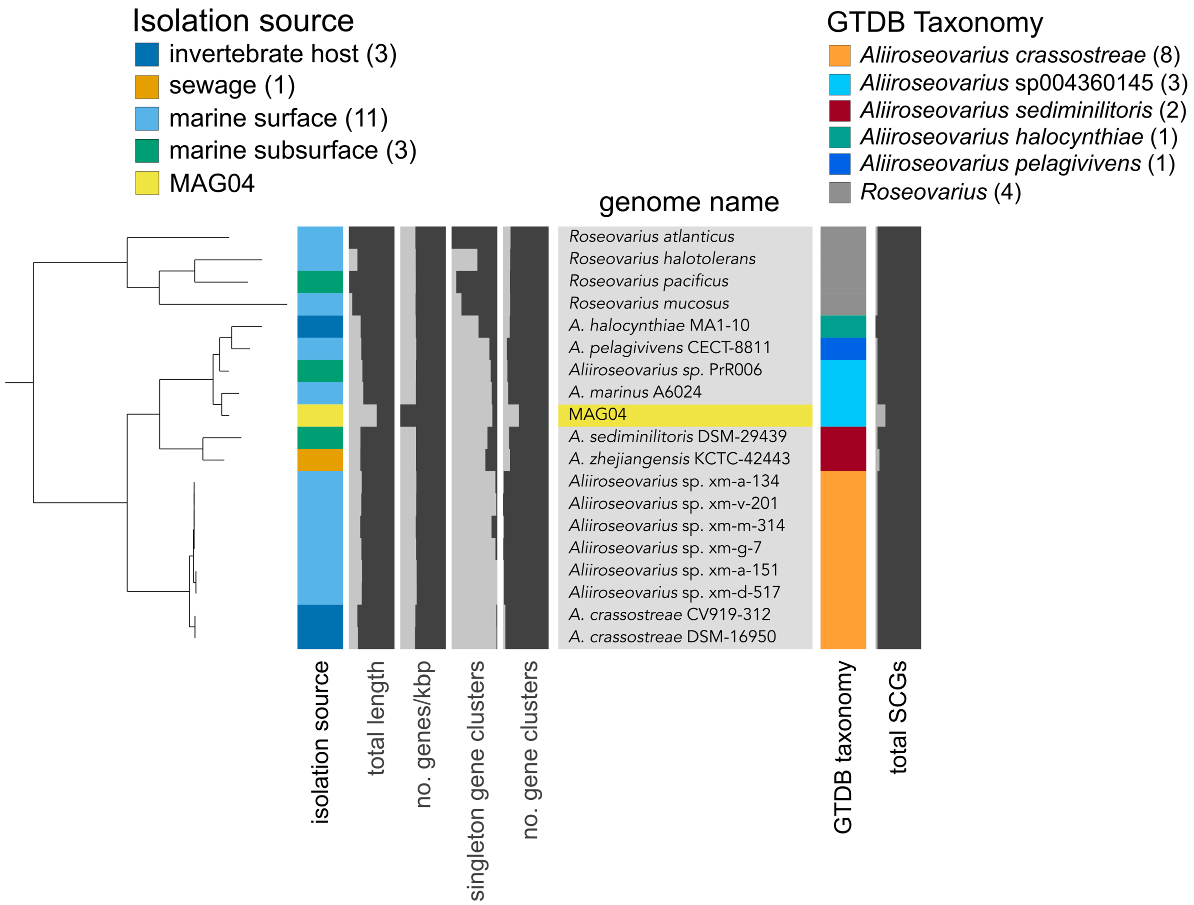

Supplementary Fig. 5. Phylogenomic analysis of MAG04. Phylogenomic placement of MAG04 against a manually curated collection of 14 *Aliiroseovarius* genomes. The tree was rooted with four *Roseovarius* genomes. The colored bar on the left indicates the broad habitat category, assigned based on the host and isolation source retrieved from NCBI's BioSample database for each genome. Values in parentheses indicate the number of genomes per category. Results of taxonomy estimates for each genome are shown on the right, based on a collection of 23 ribosomal genes from the Genome Taxonomy Database (GTDB). See Supplementary Data 4 for information on source data.

Supplementary Data

For this study, *Supplementary Data* are text files that were too large to include in the Supplementary Material. The individual files can be downloaded from the journal's website. Below are descriptions for each Supplementary Data item.

Supplementary Data 1

Differentially abundant ASVs. Data table of all 111 differentially abundant ASVs between hypoxic and normoxic conditions. ASVs that were enriched and more frequent in hypoxic and normoxic samples were identified using the Indicator Species Analysis (Dufrene and Legendre, 1997) with the R package labdsv (Roberts et al. 2007). Only ASVs represented by > 100 reads are included.

Description of table headers are as follows:

- **ASV** ASV name.
- **reads** Total reads in data set.
- **norm** Total reads in normoxic samples.
- **hypo** Total reads in hypoxic samples.
- **group** Sample group ASV is enriched in.
- **indval** Indicator value from Dufrene-Legendre Indicator Species Analysis.
- **pval** p-value from Dufrene-Legendre Indicator Species Analysis
- **freq** Total number of samples where ASV was detected.
- The remaining columns contain lineage information for each ASV.

Supplementary Data 2

KEGG module estimations for MAGs. Results of reconstructing metabolic pathways and estimating pathway completeness using anvi-estimate-metabolism for MAGs 01–05 and Bin13. All modules are included in the table, regardless of module completeness.

Description of table headers are as follows:

- **unique__id**
- **genome__name**: MAGs 01–05 and Bin13
- **kegg__module**: KEGG module number, for example M00595 is the module number for thiosulfate oxidation by SOX complex.
- **module__name**: KEGG module name.
- **module__class**: KEGG module class.
- **module__category**: KEGG module category.
- **module__subcategory**: KEGG module subcategory.
- **module__definition**: List of KOfams belonging to KEGG module.
- **module__completeness**: Percent module completeness estimated based on number of hits in **module definition**.

- **module__is__complete**: Module completeness cutoff set at 0.7, where greater value
- **kofam__hits__in__module**: KOfams found in each genome.
- **gene__caller__ids__in__module**: Corresponding gene IDs for KOfams found in each genome.

Supplementary Data 3

Metadata of genomes in MAG02 phylogenomic analysis. Metadata record of all genomes used in phylogenomic analysis of MAG02 (Supplementary Figure 4). Table contains information for each of the 72 *Arcobacter*-like genomes (plus 3 outgroup genomes).

Description of table headers are as follows:

- **genome__id**: Name of genome in phylogenomic tree.
- **isolation__category**: Broad category based on host/isolation source information retrieved from the BioSample record.
- **organism**: Organism name retrieved from the BioSample record.
- **strain__or__isolate**: Genome stain or isolate designation.
- **sample__information**: Host/isolation source information retrieved from the BioSample record.
- **bioproject**: NCBI BioProject ID.
- **biosample**: NCBI BioSample ID.
- **assembly__accession**: NCBI Assembly ID.
- **taxid**: NCBI Taxonomy ID.
- **species__taxid**: NCBI Species Taxonomy ID.
- **doi__publication**: DOI link for related publication.
- **assembly__level**: Assembly level.
- **seq__rel__date**: Genome release data.
- **ftp__path**: FTP link to download genome.
- **submitter**: Submitting Institution.

Supplementary Data 4

Metadata of genomes in MAG04 phylogenomic analysis. Metadata record of all genomes used in phylogenomic analysis of MAG04 (Supplementary Figure 5). Table contains information for each of the 14 *Aliiroseovarius* genomes (plus 4 outgroup genomes).

Description of table headers are as follows:

- **genome__id**: Name of genome in phylogenomic tree.
- **isolation__category**: Broad category based on host/isolation source information retrieved from the BioSample record.
- **organism**: Organism name retrieved from the BioSample record.
- **strain__or__isolate**: Genome stain or isolate designation.

- **sample_information**: Host/isolation source information retrieved from the BioSample record.
- **bioproject**: NCBI BioProject ID.
- **biosample**: NCBI BioSample ID.
- **assembly_accession**: NCBI Assembly ID.
- **taxid**: NCBI Taxonomy ID.
- **species_taxid**: NCBI Species Taxonomy ID.
- **doi_publication**: DOI link for related publication.
- **assembly_level**: Assembly level.
- **seq_rel_date**: Genome release data.
- **ftp_path**: FTP link to download genome.
- **submitter**: Submitting Institution.

Have a nice day.
