# Supplementary material for: Rapid ecosystem-scale consequences of acute deoxygenation on a Caribbean coral reef
Source: Nat Commun. 2021 Jul 26;12:4522. doi: 10.1038/s41467-021-24777-3 (PMC8313580; doi:10.1038/s41467-021-24777-3)
Supplement: Supplementary file 3 — Description of Additional Supplementary Files [file 41467_2021_24777_MOESM3_ESM.docx]

**Description of Additional Supplementary Files**

**Supplementary Data 1. Differentially abundant ASVs.**

Data table of all 111 differentially abundant ASVs between hypoxic and normoxic conditions. ASVs that were enriched and more frequent in hypoxic and normoxic samples were identified using the Indicator Species Analysis (Dufrene and Legendre, 1997) with the R package *labdsv* (Roberts et al. 2007). Only ASVs represented by > 100 reads are included.

**Supplementary Data 2. KEGG module estimations for MAGs.**

Results of reconstructing metabolic pathways and estimating pathway completeness using anvi-estimate-metabolism for MAGs 01–05 and Bin13. All modules are included in the table, regardless of module completeness.

**Supplementary Data 3. Metadata of genomes in MAG02 phylogenomic analysis.**

Metadata record of all genomes used in phylogenomic analysis of MAG02 (Supplementary Figure 4). Table contains information for each of the 72 *Arcobacter*-like genomes (plus 3 outgroup *Sulfurimonas* genomes).

Description of table headers are as follows:

-genome_id: Name of genome in phylogenomic tree.

-isolation_category: Broad category based on host/isolation source information retrieved from the BioSample record.

-organism: Organism name retrieved from the BioSample record.

-strain_or_isolate: Genome stain or isolate designation.

-sample_information: Host/isolation source information retrieved from the BioSample record.

-bioproject: NCBI BioProject ID.

-biosample: NCBI BioSample ID.

-assembly_accession: NCBI Assembly ID.

-taxid: NCBI Taxonomy ID.

-species_taxid: NCBI Species Taxonomy ID.

-doi_publication: DOI link for related publication.

-assembly_level: Assembly level.

-seq_rel_date: Genome release data.

-ftp_path: FTP link to download genome.

-submitter: Submitting Institution.

**Supplementary Data 4. Metadata of genomes in MAG04 phylogenomic analysis.**

Metadata record of all genomes used in phylogenomic analysis of MAG04 (Supplementary Figure 5). Table contains information for each of the 14 *Aliiroseovarius* genomes (plus 4 outgroup genomes).

Description of table headers are as follows:

-genome_id: Name of genome in phylogenomic tree.

-isolation_category: Broad category based on host/isolation source information retrieved from the BioSample record.

-organism: Organism name retrieved from the BioSample record.

-strain_or_isolate: Genome stain or isolate designation.

-sample_information: Host/isolation source information retrieved from the BioSample record.

-bioproject: NCBI BioProject ID.

-biosample: NCBI BioSample ID.

-assembly_accession: NCBI Assembly ID.

-taxid: NCBI Taxonomy ID.

-species_taxid: NCBI Species Taxonomy ID.

-doi_publication: DOI link for related publication.

-assembly_level: Assembly level.

-seq_rel_date: Genome release data.

-ftp_path: FTP link to download genome.

-submitter: Submitting Institution.
