# Supplementary material for: Rapid ecosystem-scale consequences of acute deoxygenation on a Caribbean coral reef
Source: Nat Commun. 2021 Jul 26;12:4522. doi: 10.1038/s41467-021-24777-3 (PMC8313580; doi:10.1038/s41467-021-24777-3)
Supplement: Supplementary file 8 — Reporting Summary [file 41467_2021_24777_MOESM8_ESM.pdf]

nature research

Corresponding author(s): Maggie Johnson

Last updated by author(s): 6/18/2021

Reporting Summary

Nature Research wishes to improve the reproducibility of the work that we publish. This form provides structure for consistency and transparency in reporting. For further information on Nature Research policies, see our Editorial Policies and the Editorial Policy Checklist.

Statistics

For all statistical analyses, confirm that the following items are present in the figure legend, table legend, main text, or Methods section.

n/a	Confirmed	
[ ]	[x]	The exact sample size (*n*) for each experimental group/condition, given as a discrete number and unit of measurement
[ ]	[x]	A statement on whether measurements were taken from distinct samples or whether the same sample was measured repeatedly
[ ]	[x]	The statistical test(s) used AND whether they are one- or two-sided<br>*Only common tests should be described solely by name; describe more complex techniques in the Methods section.*
[x]	[ ]	A description of all covariates tested
[ ]	[x]	A description of any assumptions or corrections, such as tests of normality and adjustment for multiple comparisons
[ ]	[x]	A full description of the statistical parameters including central tendency (e.g. means) or other basic estimates (e.g. regression coefficient) AND variation (e.g. standard deviation) or associated estimates of uncertainty (e.g. confidence intervals)
[ ]	[x]	For null hypothesis testing, the test statistic (e.g. *F*, *t*, *r*) with confidence intervals, effect sizes, degrees of freedom and *P* value noted<br>*Give P values as exact values whenever suitable.*
[x]	[ ]	For Bayesian analysis, information on the choice of priors and Markov chain Monte Carlo settings
[x]	[ ]	For hierarchical and complex designs, identification of the appropriate level for tests and full reporting of outcomes
[x]	[ ]	Estimates of effect sizes (e.g. Cohen's *d*, Pearson's *r*), indicating how they were calculated

Our web collection on statistics for biologists contains articles on many of the points above.

Software and code

Policy information about availability of computer code

Data collection	CoralNet (https://coralnet.ucsd.edu) was used for collecting data on benthic cover in permanent photoquadrats.
Data analysis	Coral response variables were analyzed in R (v4.0.2) using the packages lme4 (v1.1-26) and lmerTest (v3.1-3).<br><br>Coral benthic data were analyzed in R (v4.0.2) with the package vegan (v2.5-7).<br><br>16S rRNA data were processed and analyzed in R (v3.6.2) using DADA2 (v1.16.0), phyloseq (v1.36.0), and labdsv (v2.0-1). Primary visualization was conducted in anvi'o (v6.2-master).<br><br>Metagenomic data were preprocessed using Trimmomatic (v0.39). Trimmed data were then processed and analyzed in anvi'o (v6.2-master) using a Snakemake (v5.10.0) workflow that incorporated Illumina Utils (v2.11), MEGAHIT (v1.2.9), Prodigal (v2.6.3), KrakenUniq (v0.5.8), Bowtie2 (v2.3.5), SAMtools (v1.10), tRNAscan-SE (v2.0.5), DIAMOND (v0.9.14), MUSCLE (v3.8.1551). We also used Kaiju (v1.7.3), VirSorter (v1.0.6), CONCOCT (v1.1.0), ncbi-genome-download (v0.3.0), IQ-TREE (v2.0.3).

For manuscripts utilizing custom algorithms or software that are central to the research but not yet described in published literature, software must be made available to editors and reviewers. We strongly encourage code deposition in a community repository (e.g. GitHub). See the Nature Research guidelines for submitting code & software for further information.

Data

Policy information about availability of data

All manuscripts must include a data availability statement. This statement should provide the following information, where applicable:

- Accession codes, unique identifiers, or web links for publicly available datasets
- A list of figures that have associated raw data
- A description of any restrictions on data availability

Data Availability
Trimmed 16S rRNA (primers removed) sequence data generated in this study have been deposited in the European Nucleotide Archive (ENA) under Project Accession number PRJEB36632 (ERP119845) [https://www.ebi.ac.uk/ena/browser/view/PRJEB36632], sample accession numbers ERS4291994-ERS4292031 [https://www.ebi.ac.uk/ena/browser/view/ERS4291994-ERS4292031]. Raw 16S rRNA fastq files can be accessed through the Smithsonian figshare at doi:10.25573/data.11819745 [https://doi.org/10.25573/data.11819745]. The metagenomic sequence data generated in this study have been deposited in the ENA under Project Accession number PRJEB36632 (ERP119845) [https://www.ebi.ac.uk/ena/browser/view/PRJEB36632], sample accession numbers ERS4578390-ERS4578393 [https://www.ebi.ac.uk/ena/browser/view/ERS4578390-ERS4578393]. Related data and data products for individual analysis workflows are available through the Smithsonian figshare under the collection at doi:10.25573/data.c.5025362 [https://doi.org/10.25573/data.c.5025362.v1].

Code Availability

All code, reproducible workflows, and further information on data availability can be found on the project website at https://hypocolypse.github.io/. The code embedded in the website is available on GitHub [https://github.com/hypocolypse/web] in R Markdown format. The version of code used in this study is archived under Hypocolypse Workflows v1.0 [https://github.com/hypocolypse/web/releases/tag/v1.0], DOI identifier, doi:10.5281/zenodo.4940132 [https://doi.org/10.5281/zenodo.4940132].

Field-specific reporting

Please select the one below that is the best fit for your research. If you are not sure, read the appropriate sections before making your selection.

☐ Life sciences ☐ Behavioural & social sciences ☒ Ecological, evolutionary & environmental sciences

For a reference copy of the document with all sections, see nature.com/documents/nr-reporting-summary-flat.pdf

Ecological, evolutionary & environmental sciences study design

All studies must disclose on these points even when the disclosure is negative.

Study description

Coral reefs in Bocas del Toro, Panama were evaluated before, during, and one year after an acute deoxygenation event that occurred in September 2017. Water quality was evaluated during the event for dissolved oxygen, temperature, salinity, and pH, among other variables. Corals were collected for physiological analyses, water samples were collected from just above the coral benthos for microbial communities, and benthic community structure was evaluated through benthic photoquadrat surveys.

Research sample

Environmental conditions were measured just above the benthos at 83 sampling stations across Bahia Almirante in Bocas del Toro, Panama. These sites were selected to span the full geographical range of Bahia Almirante, and constrained to the number of sites that could be visited and sampled in the same day with two small research teams.

Agaricia tenuifolia is a common and representative Caribbean coral species that dominate shallow coral reefs where the deoxygenation event occurred. Fragments of the coral Agaricia tenuifolia were collected from 6 distinct colonies at each of two sites, one hypoxia impacted site (Cayo Roldan) and one non-impacted reference site (Cayo Coral). From each colony, three fragments were collected. Coral colonies were selected haphazardly, but only colonies with living tissue were selected. Photophysiology was measured with a PAM fluorometer from 3 points located 1 cm from the growing edge of the coral, and those values were averaged for subsequent analyses. Each fragment was destructively sampled for symbiont densities and photosynthetic pigment content. Responses of this common coral are representative of the species and other common corals that populate shallow coral reefs throughout the Caribbean.

Seawater was collected from less than 1 m above the coral benthos, with 3 samples for each site and time point (during and after the event). This sampling methods is an approach for sampling the integrated ecosystem microbial community (rather than one associated specifically with a coral species),

Benthic community structure was evaluated in 11 permanent benthic photoquadrats (0.7 x 1m2) at each site before, during, and one year after the hypoxic event.

Sampling strategy

Three fragments from 6 distinct colonies of Agaricia tenuifolia were collected from the impacted and non-impacted sites, with each fragment measuring ~4 cm2. No analyses were conducted to determine the optimal number of samples. The number of replicates were chosen to minimize error introduced by intracolony variation, and the number of colonies selected was to reduce intercolony variation. A sample site of 6 colonies is an optimal number of replication while conforming to time constraints associated with both sample and data collection.

	Three measurements for PAM fluorometry were taken per fragment to account for variation within an individual, and then averaged for subsequent analyses. This number of measurements per fragment minimizes error and maximizes accuracy of our photophysiology measurements.<br><br>The number of seawater sample replicates was chosen to provide a representative sample of the benthic-pelagic water microbial community, but also constrained by the costs associated with metagenome sequencing.<br><br>Plots within a site are considered replicates for benthic surveys. The number of plots and length of transect, as well as number of points within a photoquadrat, are standard protocols for evaluating benthic community structure and yield representative of the broader community structure at a given site.
Data collection	Environmental conditions were measured with a YSI multiparameter sonde with an optical dissolved oxygen probe (YSI EXO2 and EXO optical DO smart sensor, Yellow Springs, USA). Data were collected by NL and a team of trained researchers from the Bocas del Toro Research Station. Corals were collected and measured by MDJ, LMRB, and WW. Corals were evaluated for photophysiology with a Pulse Amplitude Modulated Fluorometer (Junior PAM), and then coral tissue was removed for symbiont counts and pigment extractions. Water samples were filtered through 0.22 um filters (Millipore), and DNA was extracted from filters with a Qiagen Powersoil extraction kit.
Timing and spatial scale	The hypoxic event was first detected in September 2017. Corals were collected from each site on Sept. 25, 2017 between 0600-1000 hrs, dark adapted for two hours, and then measured for maximum quantum yield. Coral tissue was immediately stripped and then frozen for subsequent analyses. Water samples were collected on Sept. 25, 2017 at the time of coral collection, kept on ice and in the dark until return to the lab, and then filtered immediately. Filters were frozen until DNA extractions. Benthic photoquadrat surveys were conducted four months before the event, during the event, and then one year after the event with photographs.<br><br>The timing of data collection was critical because we needed to capture the event in progress. The duration of hypoxia events in situ is unknown, particularly in the tropics, so a rapid response to the detected event was critical. The follow-up sampling allowed evaluation of immediate responses to the event and a one-year recovery period (for the benthos).<br><br>The spatial scale sample spanned from the impacted hypoxic site to a reference normoxic site, selected specifically to analyze responses and conditions associated with the event in relation to unaffected sites. Both sites are within the Bocas del Toro archipelago, and are approximately 30 km apart.
Data exclusions	No data were excluded from analyses.
Reproducibility	Multiple measurements were taken at each site and sampling station for reproducibility, and then averaged for analyses. Coral fragments were measured in three adjacent spots for photophysiology to account for variation within an individual, and then averaged for each individual for subsequent analyses. Due to the unique event, and unpredictable nature, we were unable to replicate this experiment. There have been no other events in the area since this event in 2017.
Randomization	Water samples were collected from randomly selected, stratified sampling stations. Water samples were collected haphazardly from above the benthos were coral colonies were samples. Coral colonies were selected haphazardly from live colonies at each site. Permanent photoquadrats were randomly installed before the event, with quadrats placed every 5 m along a 50 m transect. Plots were then permanent marked and resampled over time.<br><br>Two sites were specifically selected to represent the hypoxic, impacted site and the normoxic, reference site. Beyond those specific experimental groups, all replicates were collected randomly or haphazardly within a set of experimental constraints (to reduce the impact of confounding factors). Coral fragments were collected from individual colonies that were located >10 m apart, to ensure genotypic differences in the absence of genetic sequencing. Living colonies at the depth limit of the study (the oxycline at a depth of 3-4 m) were selected haphazardly, and replicate fragments within colonies were collected from unshaded branch tips, haphazardly. Covariates were controlled by collecting samples from the same depth and the same light environment (for corals).
Blinding	All samples were marked with codes to allow for blind data collection. The people performing measurements did not know what samples came from the impacted vs. non-impacted sites during data collection.

Did the study involve field work? ☒ Yes ☐ No

Field work, collection and transport

Field conditions	Environmental conditions were measured during the hypoxic event and values are presented in the main text of the manuscript and the supplemental information. At each sampling point (before, during, or after), all measurements for that period were taken on the same day and effort was made to reduce the time between at each site.<br><br>Environmental conditions measured during the study were dissolved oxygen concentrations, salinity, temperature, pH, and chlorophyll concentrations in RFU (relative fluorescent units).
Location	Two sites used in this study for coral and water column sampling were Cayo Roldan (9.22023, -82.3231) and Cayo Coral (9.254583, -82.125383) in Bocas del Toro, Panama. Water samples were collected just above the benthos (0.5 m) at a depth of 3-5 m, and coral samples were collected from a depth of 3-5 m.
Access & import/export	Samples were collected and analyzed within the country of Panama at the Smithsonian Tropical Research Institute. Coral and water samples were collected under a permit from the Ministerio de Ambiente, Republic de Panama (No.SE/A-124-16) to AHA and MDJ.
Disturbance	Water collection caused no disturbance to the environment. Coral fragments were collected by carefully removing three small

fragments from each coral colony. Impact was minimized by selecting living corals. Corals can reproduce via asexual fragmentation and can recover from damage so impact of coral collection was minimal. Permanent quadrats had minimal impact on the habitat because they are photographed from ~1 m above the benthos, and plots are marked by PVC poles placed into loose substrate (not living coral).

Reporting for specific materials, systems and methods

We require information from authors about some types of materials, experimental systems and methods used in many studies. Here, indicate whether each material, system or method listed is relevant to your study. If you are not sure if a list item applies to your research, read the appropriate section before selecting a response.

Materials & experimental systems

n/a	Involved in the study
x	Antibodies
x	Eukaryotic cell lines
x	Palaeontology and archaeology
	x Animals and other organisms
x	Human research participants
x	Clinical data
x	Dual use research of concern

Methods

n/a	Involved in the study
x	ChIP-seq
x	Flow cytometry
x	MRI-based neuroimaging

Animals and other organisms

Policy information about studies involving animals; ARRIVE guidelines recommended for reporting animal research

Laboratory animals	This study did not involve laboratory animals.
Wild animals	Agaricia tenuifolia is one of the dominant corals on the shallow coral reefs of Bahia Almirante, Bocas del Toro, Panama. Coral fragments were collected by carefully removing three small fragments from each coral colony with bone cutters. Fragments were then stored in seawater briefly for transport to the lab. After photophysiology measurements coral tissue was immediately removed. Age of these corals is indeterminate due to their clonal nature and propagation through asexual fragmentation.<br><br>Corals were sampled alive for photophysiology and then destructively sampled for symbiont densities and pigment content (the only method for collecting that data).
Field-collected samples	Corals were maintained in a cooler with replenished seawater immediately after collection. Corals were stored in the dark to allow for dark adaptation prior to photophysiology measurements. Corals were maintained in the coolers for no more than 4 hours before measurements were taken. Directly after measurements were taken for photophysiology the coral tissue was stripped from the underlying skeleton and frozen at -20C for subsequent analyses.
Ethics oversight	Corals are invertebrates and no ethics oversite was required. We regularly consult with the Ministerio de Ambiente, Republic de Panama to ensure that we minimize damage to corals and the surrounding habitat.

Note that full information on the approval of the study protocol must also be provided in the manuscript.
